# Evidences for the involvement of monoaminergic and GABAergic systems in antidepressant-like activity of garlic extract in mice

**DOI:** 10.4103/0253-7613.43165

**Published:** 2008-08

**Authors:** Dinesh Dhingra, Vaibhav Kumar

**Affiliations:** Pharmacology Division, Department of Pharmaceutical Sciences, Guru Jambheshwar University of Science and Technology, Hisar, Haryana, India

**Keywords:** *Allium sativum*, depression, forced swim test, monoamine oxidase, tail suspension test

## Abstract

**Objectives:**

The present study was undertaken to investigate the effect of the ethanolic extract of *Allium sativum*  L. (Family: Lilliaceae), commonly known as garlic, on depression in mice.

**Materials and Methods:**

Ethanolic extract of garlic (25, 50 and 100 mg/kg) was administered orally for 14 successive days to young Swiss albino mice of either sex and antidepressant-like activity was evaluated employing tail suspension test (TST) and forced swim test (FST). The efficacy of the extract was compared with standard antidepressant drugs like fluoxetine and imipramine. The mechanism of action of the extract was investigated by co-administration of prazosin (α1-adrenoceptor antagonist), sulpiride (selective D2-receptor antagonist), baclofen (GABA_B_  agonist) and p-CPA (serotonin antagonist) separately with the extract and by studying the effect of the extract on brain MAO-A and MAO-B levels.

**Results:**

Garlic extract (25, 50 and 100 mg/kg) significantly decreased immobility time in a dose-dependent manner in both TST and FST, indicating significant antidepressant-like activity. The efficacy of the extract was found to be comparable to fluoxetine (20 mg/kg p.o.) and imipramine (15 mg/kg p.o.) in both TST and FST. The extract did not show any significant effect on the locomotor activity of the mice. Prazosin, sulpiride, baclofen and p-CPA significantly attenuated the extract-induced antidepressant-like effect in TST. Garlic extract (100 mg/kg) administered orally for 14 successive days significantly decreased brain MAO-A and MAO-B levels, as compared to the control group.

**Conclusion:**

Garlic extract showed significant antidepressant-like activity probably by inhibiting MAO-A and MAO-B levels and through interaction with adrenergic, dopaminergic, serotonergic and GABAergic systems.

## Introduction

Depression is a common mood disorder that affects a person's life, affecting his/her mood, thoughts, thinking, behavior, feelings etc. The main biochemical theory of depression is the monoamine hypothesis, which states that depression is caused by a functional deficit of monoamines (norepinephrine, serotonin and dopamine) at certain sites in brain.[1] Reduced monoaminergic signaling has long been thought to underlie depressive disorders. For example, reduced monoamine metabolite levels have been found in the cerebrospinal fluid of depressed individuals; likewise, serotonin (5-HT), norepinephrine or dopamine depletion exerts pro-depressive effects.[2–3] Monoamine oxidase (MAO) is responsible for metabolizing monoamines like norepinephrine, dopamine and 5-HT. MAO is found in nearly all tissues and exists in two forms: MAO-A and MAO-B. MAO-A has substrate preference for serotonin and is the main target for the antidepressant monoamine oxidase inhibitors. MAO-B   has substrate preference for phenylethyl amine. Both enzymes act on noradrenaline and dopamine. In the case of depression, the level of monoamine oxidase enzyme in the brain is increased, which in turn reduces the levels of monoamines.[4–5] Advances made in diverse areas of neuroscience suggest that the neurotransmitter system, in addition to the monoamines, contributes to the pathophysiology of depression. Acute and chronic treatment with CGP56433A, a selective GABA_B_  receptor antagonist, decreased immobility in FST. Thus, GABA_B_  receptor antagonism may serve as a basis for the generation of novel antidepressants.[6] GABA levels have been reported to be decreased in the cerebrospinal fluid of depressed patients, in some studies.[7–8] In rats, antidepressants and mood stabilizers appear to up-regulate frontal-cortical GABA_b_  but not GABA_a_  receptors.[9–10]

Since all the synthetic drugs available for the treatment of depression have various adverse effects and problematic interactions, our aim was to explore the potential of plants in the management of depression. *Allium sativum*  Linn. (Family: Lilliaceae) is commonly known as garlic. Dried bulbs of garlic are incorporated in day to day practice as condiment/spice in the food items. Garlic is endowed with several medicinal properties.[11] It has been reported to possess anti-stress,[12] anti-ageing, memory improving properties and has the potential for preventing the progression of Alzheimer's disease.[13] The antidepressant property of this plant has not been reported scientifically. Therefore, our study was focused on evaluation of the antidepressant potential of *Allium sativum*  in mice and to also study probable mechanisms responsible for the antidepressant activity.

## Materials and Methods

### Collection of Plant material

Dried bulbs of *Allium sativum * Linn. were purchased from the local market in Hisar, Haryana (India).

### Preparation of extract of Allium sativum

About 200 g of chopped garlic slices were imbibed in 20% v/v ethanol for 10 days, at ambient temperature (37^°^ C) and filtered. The crude extract was dried on a water bath and kept in a refrigerator till further use. The yield of the extract was 20% w/w. Garlic extract has been reported to contain active components like allicin, alliin, allyl methyl thiosulfinate, γ-glutamyl-S-allyl cysteine, γ-glutamyl-S-cis-1-propenyl cysteine.[14]

### Animals

Swiss albino mice of either sex, three to four months of age and weighing around 20-30 g were procured from the Disease Free Small Animal House, CCS Haryana Agricultural University, Hisar (Haryana), India. The animals had free access to food and water. However, food was withdrawn 1 h before and 2 h after the administration of the drugs. The animals were housed in an animal room, with alternating light-dark cycle of 12 hr each. The animals were acclimatized to the laboratory conditions for at least five days prior to the behavioral experiments. The experiments were carried out between 0900 h and 1800 h. The Institutional Animal Ethics Committee (IAEC) approved the experimental protocol. Care of laboratory animals was in adherence with the guidelines specified by the CPCSEA, Ministry of Forests and Environment, Government of India (Registration number 0436).

### Drugs and chemicals

Fluoxetine hydrochloride (FLUDAC, Cadila Pharmaceuticals, Ahmedabad, India); (±) sulpiride, prazosin hydrochloride, DL-p-chlorophenyl alanine (p-CPA), imipramine hydrochloride, baclofen (Sigma-Aldrich, St. Louis, U.S.A); acetic acid, chloroform and tris (s.d. Fine Chemicals, Mumbai, India), EDTA (Hi Media Laboratories, Mumbai, India); sucrose, disodium hydrogen phosphate and sodium hydroxide (CDH, New Delhi) were used in present study.

### Vehicles

The extract was dissolved in distilled water. Fluoxetine, imipramine, prazosin and baclofen were dissolved separately in normal saline (0.9%). Sulpiride was dissolved in normal saline, followed by the addition of one drop of glacial acetic acid. p-Chlorophenylalanine (p-CPA) was dissolved in a minimum quantity of 0.1 N sodium hydroxide and the pH was adjusted to 7 with 0.1 N hydrochloric acid. The volume for oral administration and intraperitoneal injection was 1 ml/100 g of mouse.

### Laboratory Models employed for Testing Antidepressant activity

Forced-swim test: Forced swim test was proposed as a model to test antidepressant activity by Porsolt *et al.*[15] The method was the same as described by Dhingra and Sharma[16] Mice were forced to swim individually in a glass jar (25 × 12 × 25 cm^3^) containing fresh water up to 15 cm height and maintained at 25^°^ C (± 3^°^ C).[17] After an initial 2 min period of vigorous activity, each animal assumed a typical immobile posture. A mouse was considered to be immobile when it remained floating in the water without struggling, making only minimum movements of its limbs, necessary to keep its head above the water. The total duration of immobility was recorded during the next 4 min of the total test duration of six minutes. The changes in immobility duration were studied after administrating the drugs in separate groups of animals. Each animal was used only once.

*Tail-suspension test: * The total duration of immobility induced by tail suspension was measured according to the method described as a means of evaluating potential antidepressants.[18] Mice were suspended on the edge of a table, 50 cm above the floor, with the help of an adhesive tape placed approximately 1 cm from the tip of the tail. Immobility time was recorded during a 6 min period. The animal was considered to be immobile when it did not show any movement of the body and hanged passively. 

### Measurement of MAO-A and MAO-B activities

On the 14^th^  day, the mice were sacrificed after 6 min exposure to FST, and the brain samples were collected immediately on an ice plate. The collected brain samples were washed with cold 0.25M Sucrose-0.1M Tris-0.02M EDTA buffer (pH 7.4) and weighed. The whole procedure of brain isolation was completed within five minutes.[19–20]

Mouse brain mitochondrial fractions were prepared following the procedure of Schurr and Livne.[19] The MAO activity was assessed spectrophotometrically, with slight modifications.[20–22] The protein concentration was estimated by Lowry method, using bovine serum albumin as the standard.[23]

### Measurement of Locomotor activity

The locomotor activity of the control group and the animals treated with the extract was evaluated with the help of Photoactometer (INCO, Ambala, India). The differences in the locomotor activity scores were noted before and after the drug treatment.

### Experimental protocols

The animals were divided into 24 groups and each group comprised a minimum of six mice.

### Groups for Tail Suspension Test (TST)

Group 1:Control group (vehicle for garlic extract): Distilled water was administered orally for 14 successive days. At 90 min after administration on the 14^th^  day, immobility period was recorded.

Group 2: Fluoxetine (20 mg/kg) was administered orally for 14 successive days. At 90 min after administration on the 14^th^  day, immobility period was recorded.

Group 3: Imipramine (15 mg/kg) was administered orally for 14 successive days. At 90 min after administration on the 14^th^  day, immobility period was recorded.

Group 4, 5 and 6: Garlic extract (25, 50 and 100 mg/kg respectively) was administered orally for 14 successive days. At 90 min after administration on the 14^th^  day, immobility period was recorded.

### Groups for Forced Swim Test (FST)

Groups 7 to 12 are the same as groups 1 to 6 mentioned in the groups for TST, except that immobility period was recorded using FST.

### Groups for studying the mechanism of action of garlic extract

Group 13 (Control Sulpiride): Distilled water was administered orally for 14 successive days and then sulpiride (50 mg/kg) was injected on the14^th^  day, after 45 min of last oral administration of vehicle. The animals were subjected to TST, after 45 min of sulpiride injection.

Group 14: Garlic extract (100 mg/kg) was administered orally for 14 successive days and then sulpiride (50 mg/kg) was injected on the 14^th^  day, after 45 min of last oral administration of extract. The animals were subjected to TST, after 45 min of sulpiride injection.

Group 15 (Control Prazosin): Distilled water was administered orally for 14 successive days and then prazosin (62.5 *µ*g/kg) was injected on the 14^th^  day, after 45 min of last oral administration of vehicle. The animals were subjected to TST, after 45 min of prazosin injection.

Group 16: Garlic extract (100 mg/kg) was administered orally for 14 successive days and then prazosin (62.5 *µ*g/kg) was injected on the 14^th^  day, after 45 min of last oral administration of extract. The animals were subjected to TST, after 45 min of Prazosin injection.

Group 17 (Control p-CPA): Distilled water was administered orally for 14 successive days and then p-CPA (100 mg/kg) was injected from the 11^th^  day to the 14^th^  day, after 45 min of last oral administration of vehicle. The animals were subjected to TST, after 45 min of p-CPA injection.

Group 18: Garlic extract (100 mg/kg) was administered orally for 14 successive days and then p-CPA (100 mg/kg) was injected from the 11^th^  day to the 14^th^  day, after 45 min of last oral administration of extract. The animals were subjected to TST, after 45 min of p-CPA injection.

Group 19 (Control Baclofen): Distilled water was administered orally for 14 successive days and then baclofen (10 mg/kg) was injected on the 14^th^  day, after 45 min of last oral administration of vehicle. The animals were subjected to TST after 45 min of baclofen injection.

Group 20: Garlic extract (100 mg/kg) was administered orally for 14 successive days and then baclofen (10 mg/kg) was injected on the 14^th^  day, after 45 min of last oral administration of extract. The animals were subjected to TST, after 45 min of baclofen injection.

### Groups for biochemical estimations

Group 21: Distilled water was administered orally for 14 successive days. The mice were sacrificed under light anesthesia with chloroform and the brain was dissected and used for the estimation of monoamine-oxidase A and B levels.

Group 22: Imipramine (15 mg/kg) was administered orally for 14 successive days. The mice were sacrificed under light anesthesia with chloroform. The brain was dissected and used for the estimation of monoamine-oxidase A and B levels.

Group 23: Garlic extract (100 mg/kg) was administered orally for 14 successive days. The mice were sacrificed under light anesthesia with chloroform. The brain was dissected and used for the estimation of monoamine-oxidase A and B levels.

### Groups for locomotor activity

Group 24: The effect of garlic extract (100 mg/kg) on the locomotor function of mice was studied using Photoactometer (INCO, Ambala, India), to rule out the increase in the locomotor performance of mice due to the extract. The difference in the locomotor activity scores was noted before and after administration of the extract. 

### Statistical analysis

All the results were expressed as Mean ± Standard Error (SEM). Data was analyzed using one-way anova, followed by Dunnett's t-test. The data for locomotor activity scores was subjected to paired t-test. *P*  < 0.05 was considered as statistically significant.

## Results

### Effect of garlic extract on immobility periods in  TST and  FST

Garlic extract (25, 50 and 100 mg/kg, p.o.) administered to mice for 14 successive days decreased immobility periods significantly in a dose-dependent manner, as compared to control in both TST and FST, indicating significant antidepressant-like activity. A dose of 100 mg/kg p.o. of the extract showed the most potent antidepressant effect, as indicated by the highest decrease in the immobility period. The efficacy of the extract was found to be comparable to fluoxetine (20 mg/kg) and imipramine (15 mg/kg) administered for two successive weeks in both FST and TST [Tables 1 and 2.]

**Table 1:** Effect of ethanolic extract of *Allium sativum* on immobility period in tail suspension test

*Group No.*	*Treatment for 14 days p.o.*	*Number of animals*	*Dose (kg-1)*	*Immobility time (sec.)(Mean ± SEM)*
1.	Control (vehicle treated)	6	10 ml	184.2 ± 7.1
2.	Fluoxetine	5	20 mg	82.4 ± 5.9*
3.	Imipramine	5	15 mg	78.8 ± 12.7*
4.	GE	6	25 mg	123.7 ± 7.3*
5.	GE	6	50 mg	101.8 ± 4.4*
6.	GE	6	100 mg	84.3 ± 12.5*

GE = garlic extract, Statistical analysis of data was carried out by one-way ANOVA followed by Dunnett's t-test as compared to control.

* P<0.05 as compared to control F (5, 28) = 21.48 (P <0.0001)

**Table 2:** Effect of ethanolic extract of *Allium sativum* on immobility period in forced swim test

*Group No.*	*Treatment for 14 days p.o.*	*Number of animals*	*Dose (kg-1)*	*Immobility time (sec.)(Mean ± SEM)*
7.	Control (vehicle treated)	6	10 ml	147.8 ± 8.3
8.	Fluoxetine	5	20 mg	47.2 ± 5.7*
9.	Imipramine	5	15 mg	55 ± 11.6*
10.	GE	6	25 mg	72.0 ± 5.0*
11.	GE	6	50 mg	63.7 ± 5.3*
12.	GE	6	100 mg	22.7 ± 4.1*

GE= Garlic extract, Statistical analysis of data was carried out by one-way ANOVA followed by Dunnett's t-test as compared to control.

* P<0.05 as compared to control, F (5, 28) = 40.07 (*P* < 0.0001)

### Effect of combination of garlic extract with sulpiride, prazosin, p-CPA and baclofen on immobility period in TST

Sulpiride (50 mg/kg i.p.), prazosin (62.5 mg/kg i.p.) and p-CPA (100 mg/kg i.p.) alone significantly increased the immobility period, as compared to the control group. Pretreatment of animals with sulpiride or p-CPA or prazosin or baclofen significantly blocked the decrease of immobility time elicited by garlic extract (100 mg/kg p.o.) in TST [Table 3].

**Table 3:** Effect of combination of ethanolic extract of *Allium sativum* with sulpiride, baclofen, p-CPA and prazosin

*Group No.*	*Treatment for 14 days p.o.*	*Number of animals*	*Dose (kg-1)*	*Immobility time (Sec.) (Mean ± SEM)*
1	Control (vehicle treated)	6	10 ml	184.2 ± 7.1
6	GE	6	100 mg	84.3 ± 12.5^a^
13	Vehicle + Sulpiride	6	50 mg	241 ± 6.7^a^
14	GE + Sulpiride	6	100 mg	
			+ 50 mg	157 ± 8.5^b^
15	Vehicle + Prazosin	6	62.5 μg	218.7 ± 10.4^a^
16	GE + Prazosin	6	100mg	
			+ 62.5 μg	176.9 ± 7^b^
17	Vehicle + PCPA	6	100 mg	216.9 ± 5.4^a^
18	GE + PCPA	6	100mg	
			+ 100 mg	226.3 ± 6.1^b^
19	Vehicle + Baclofen	6	10 mg	198 ± 13.1
20	GE + Baclofen	6	100 mg	
			+ 10 mg	147.9 ± 5.5^b^

GE = Garlic extract, Statistical analysis of data was carried out by one-way ANOVA followed by Dunnett's t-test. F (9, 50) = 28.966 (*P* < 0.0001).

a *P* < 0.05 as compared to control

b *P* < 0.05 as compared to Garlic extract

### Effect of Garlic extract on brain monoamine oxidase (MAO) activity

Garlic extract (100 mg/kg p.o.) administered to mice, for 14 successive days, significantly decreased brain MAO-A and MAO-B activity, as compared to the control group. MAO inhibition was comparable to imipramine [Table 4].

**Table 4:** Effect of ethanolic extract of *Allium sativum* on brain MAO-A and MAO-B activities or level

*Group No.*	*Treatment for 14 days p.o.*	*Dose (kg-1)*	*MAO-A (u/g proteins)*	*MAO-B (u/g proteins)*
21	Control	10 ml	33.97 ± 2.21	46.53 ± 2.102
22	Imipramine	15 mg	14.39 ± 1.91*	29.55 ± 3.19*
23	GE	100 mg	12.14 ± 1.96*	28.75 ± 3.27*

GE - Garlic extract, Values are in Mean ± SEM, (n = 6).

* *P*<0.05 as compared to control, Statistical analysis of data was carried out by one-way ANOVA followed by Dunnett's t-test. F (2, 15) = 34.985; P<0.0001 (for MAO-A). F (2, 15) = 11.956; *P* < 0.001 (for MAO-B).

### Effect of garlic extract on locomotor activity

Garlic extract (100 mg/kg p.o.) administered for 14 successive days did not show any significant change in the locomotor function of mice (870.1 ± 99.8), as compared to the control (805 ± 121.5) group.

## Discussion

In the present study, 20% ethanolic extract of garlic (25, 50 and 100 mg/kg p.o.) administered to mice, for 14 successive days, showed significant antidepressant-like activity in Forced Swim Test (FST) and Tail Suspension Test (TST). The efficacy of garlic extract was found to be comparable to fluoxetine and imipramine. FST and TST are two commonly used behavioral despair models of depression. These tests are quite sensitive and widely employed in rodents to predict antidepressant potential by decrease of immobility period produced by several different classes of antidepressant drugs.[15,17] It has been reported that TST is less stressful and has higher pharmacological sensitivity than FST.[23]

Garlic extract did not show significant change in the locomotor activity of mice, as compared to the control group; so this extract did not produce any motor effects. It confirms the assumption that the antidepressant-like effect of the extracts is specific. According to our results, the antidepressant-like effect of garlic extract was significantly reversed by the treatment of animals with prazosin (α_1_ -adrenoceptor antagonist), sulpiride (a selective dopamine D_2_ -receptor antagonist), p-CPA (serotonin synthesis inhibitor) and baclofen (GABA_B_  agonist) when tested in TST. This suggests that garlic extract might produce antidepressant-like effect through interaction with α_1_ -adrenoceptors, dopamine D_2_  receptors, serotonergic and GABAergic receptors, thereby increasing the levels of norepinephrine, dopamine, serotonin and decreasing GABA levels in the brain of mice. Garlic extract (100 mg/kg p.o.) administered to mice, for 14 successive days, significantly decreased brain MAO-A and MAO-B activities as compared to the control group. MAO inhibiting activity by the extract was comparable to imipramine. Thus, antidepressant-like activity of the extract might also be due to inhibition of MAO, resulting in increase in the brain levels of monoamines.

In conclusion, garlic extract showed significant antidepressant-like activity probably by inhibiting MAO-A and MAO-B levels, and through interaction with adrenergic, dopaminergic, serotonergic and GABAergic systems. The antidepressant-like action of the extract was comparable to imipramine and fluoxetine. Hence, garlic extract may have potential therapeutic value for the management of depressive disorders.
